# Air pollution mixtures and cognitive outcomes in children: associations with school-age exposure and sex differences

**DOI:** 10.1007/s00431-026-06841-6

**Published:** 2026-03-19

**Authors:** Xiruo Kou, Josefa Canals, Victoria Arija

**Affiliations:** 1 Nutrition and Mental Health (NUTRISAM) Research Group, Universitat Rovira I Virgili, Reus, 43204 Spain; 2 Institut d’Investigació Sanitaria Pere Virgili (IISPV), Reus, 43204 Spain; 3 Nutrition and Public Health Unit, C/Sant Llorenç 21, Reus, 43201 Spain

**Keywords:** Air pollution mixture, Early-life exposure, Cognitive development, Sex stratification, Weighted quantile sum

## Abstract

Children are highly vulnerable to air pollution, yet most studies rely on single-pollutant approaches. This study examined associations between individual and joint exposure to multiple air pollutants and cognitive outcomes in preschoolers, with attention to potential sex differences. Traffic-related pollutants (PM_2.5_, PM_10_, PM_coarse_, PM_2.5absorbance_, NO_2_, NO_x_, O_3_) were estimated at schools from the cross-sectional study among 286 children. Cognitive function was assessed with the Wechsler Preschool and Primary Scale of Intelligence–IV. Linear and weighted quantile sum (WQS) regression were conducted, adjusting for relevant child and family covariates. PM_coarse_ (*β* = −2.71, 95% CI −4.23, -1.20) and PM_10_ (*β* = −2.39, 95% CI −4.09, −0.70) were inversely associated with the working memory index (WMI), with stronger associations observed in boys (PM_coarse_: *β* = −3.32, 95% CI −5.77, −0.88; PM_10_: *β* = −3.79, 95% CI −6.50, −1.09). WQS analysis indicated a negative association between the pollutant mixture and WMI (estimate = − 3.60, 95% CI −5.92, −1.28), with PMcoarse contributing most to the index. Sex-stratified WQS analyses suggested associations between air pollution mixture and lower verbal comprehension in girls and lower WMI in boys.

*Conclusion*: School-age exposure to ambient air pollution mixtures was associated with differences in cognitive performance among preschool children, with patterns varying by sex. These findings highlight the importance of considering pollutant mixtures and potential sex differences when evaluating associations between air pollution and early childhood cognition.

*Clinical trial registration:* EUCTR-2012-005480-28 (www.clinicaltrialsregister.eu). NCT03196882 (www.clinicaltrials.gov).

**Table:** 

**What is Known:** *• Ambient air pollution is linked to impaired neurodevelopment in children, but evidence using multi-pollutant mixture models in preschoolers remains limited.*	
**What is New:** *• Air pollutant mixture was negatively associated with working memory index in preschoolers.* *• Sex-stratified analyses suggested potential associations with lower verbal comprehension in girls and lower working memory in boys.*

**Supplementary Information:**

The online version contains supplementary material available at 10.1007/s00431-026-06841-6.

## Introduction

Ambient air pollution has been recognized as one of the most pressing global public health concerns [1]. Beyond its well-established cardiopulmonary consequences, accumulating evidence suggests that exposure to air pollutants may also impair neurodevelopment [2, 3]. Children are particularly vulnerable to environmental toxicants because of their higher ventilation per body weight, immature detoxification systems, and the rapid pace of brain development during the early years of life [4]. Cognitive functions, which encompass core abilities such as attention, memory, executive functions, and reasoning, undergo critical development during the preschool period. These functions serve as the foundation for later academic performance, socioeconomic success, and mental health [5, 6]. Thus, understanding the impact of early-life air pollution on cognitive development is of paramount public health importance.

Epidemiological studies have increasingly examined associations between air pollution and neurodevelopmental outcomes among children. Consistent evidence links higher levels of traffic-related pollutants to poorer cognitive outcomes, including reduced intelligence quotient (IQ), memory deficits, and attention problems [7]. Studies conducted in school environments have further shown that exposure to ambient pollutants, including particulate matter ≤ 2.5 µm in diameter (PM_2.5_) and nitrogen dioxide (NO_2_), can adversely associate with children’s working memory, attention, and academic performance [8, 9]. These findings emphasize early childhood as a critical period of susceptibility for higher-order neurocognitive function [10].

Despite this growing evidence, several important limitations persist in the literature. First, the majority of studies have used single-pollutant models, even though pollutants often co-occur and are highly correlated in real-world settings [11, 12]. This approach fails to capture their combined neurotoxic effects and obscures the identification of the most harmful constituents. Second, relatively few studies have systematically assessed potential sex-specific differences, even though such differences are biologically plausible given variations in endocrine, genetic, and metabolic pathways that shape neurodevelopment, and existing results remain conflicting [13, 14].

Thus, this cross-sectional study aims to assess the association between exposure to a mixture of air pollutants—including ozone (O_3_), coarse particulate matter (PM₁₀ − PM₂.₅, PM_coarse_), particulate matter ≤ 10 µm in diameter (PM_10_), particulate matter ≤ 2.5 µm (PM₂.₅), PM_2.5_, absorbance (PM_2.5abs_), nitrogen oxides (NO_x_), and NO_2_—and children’s cognitive development in the school environment, where they spend many hours each day. Both linear regression and weighted quantile sum (WQS) regression were applied to evaluate the individual and combined associations of these pollutants. This analytical approach offers a more nuanced understanding of the complex association between air pollution exposures and cognitive function and identifies sex-specific susceptibility patterns, thereby informing targeted preventive strategies.

## Materials and methods

### Study design and participants

This study is based on the Ensayo CLInico Para Suplementar con hierro a EmbarazadaS (ECLIPSES) project, a community-based cohort in Tarragona, Catalonia, Spain, conducted between 2013 and 2017. Detailed information on the study protocol, including inclusion and exclusion criteria, has been reported elsewhere [15]. The study adhered to the Declaration of Helsinki and was approved by the Ethics Committee of the Institut d’Investigació en Atenció Primaria de Salut (IDIAP) and the Institut d’Investigació Sanitària Pere Virgili (approval ID: 118/2017; 28 September 2017). All participants provided written informed consent. The project was later extended to follow children up to age 4 to assess neurodevelopment (continuing as ECLIPSES-NEN). Analyses for the present cross-sectional study included data from 286 children, incorporating school air pollution exposure and cognitive assessments. The study flow is shown in Fig. S1.

### Assessment of air pollution exposure

Exposure levels of traffic-related air pollution at school addresses were assessed for the participating children. This assessment was conducted as part of the European Study of Cohorts for Air Pollution Effects (ESCAPE). Between January 2009 and January 2010, monitoring was carried out at 80 sites across Catalonia to measure nitrogen oxides (NO_X_ and NO_2_) [16]. Measurements were taken during three separate two-week periods covering different seasons. In addition, a subset of 40 sites was used to collect particulate matter metrics, including mass concentrations of PM_10_, PM_2.5_, and the absorbance of PM_2.5_ (PM2.5abs) [17]. The concentration of PM_coarse_ was derived as the difference between PM_10_ and PM_2.5_. To obtain robust annual average estimates for each pollutant, measurements were temporally adjusted by comparing sampling-period concentrations with annual averages from a continuous reference station and applying the resulting difference to correct the measured values. Subsequently, land use regression (LUR) models were developed incorporating multiple geographic predictor variables [18]. These models were applied to estimate pollution levels at school addresses of the study participants, providing address-level estimates. The LUR models demonstrated good predictive performance, with R^2^ values of 62% for PM_2.5_, 76% for PM_10_, 76% for PM_coarse_, 75% for PM_2.5abs_, 71% for NO_2_, and 69% for NO_X_. For O_3_, we applied the Europe-wide LUR model developed by the Effects of Low-Level Air Pollution: A Study in Europe (ELAPSE) project for the reference year 2010 [19], which provided continuous concentration surfaces at a 100 m × 100 m grid resolution. Temporal refinement using daily baseline measurements from a local background monitoring station was applied to align exposures with the actual school attendance periods, yielding daily pollution exposure estimates. For pollutants without pollutant-specific daily monitoring data, these estimates were considered semi-quantitative rankings of exposure levels rather than absolute concentration measurements. A schematic overview of the temporal adjustment and exposure assignment process is shown in Fig. S2.

Because both ESCAPE and ELAPSE models provide spatial exposure estimates for a single reference year (2009–2010 for ESCAPE and 2010 for ELAPSE), temporal extrapolation was applied to align exposure estimates with the exact timing of each participant’s school attendance period. Following the standard ESCAPE ratio method, daily pollutant concentrations were estimated by combining the spatial LUR estimates with temporal trends derived from routine background monitoring stations. Specifically, the daily exposure concentration for a given pollutant was calculated by multiplying its spatial LUR estimate at the school address (for the reference year) by a daily adjustment ratio. This ratio was derived from routine monitoring stations by dividing the daily average concentration by the annual average concentration from the reference year, with the final ratio representing the average across all selected stations.

Routine monitoring stations were selected based on predefined criteria: location within a 100 km buffer of the study area, classification as background stations, availability of data during the reference year, and availability of data across the full study period (2001–2018), with less than 25% missing data at both daily (≤ 6 missing hours per day) and annual levels (≤ 25% missing daily data per year). After analyzing data availability from different sources, NO₂ ratios were calculated using monitoring stations from the Spanish national dataset, while O₃ ratios were calculated using stations from the Catalan regional dataset due to more complete temporal coverage.

For pollutants not routinely measured at monitoring stations (NO_X_, PM_2.5abs_, PM_coarse_), as well as for PM_2.5_ and PM_10_ when complete temporal coverage was unavailable, temporal extrapolation was based on daily NO_2_ ratios, consistent with established ESCAPE procedures [16]. This approach assumes similar temporal patterns between these pollutants, which is supported by their common traffic-related sources [20, 21].

Daily exposures were then aggregated over each academic year (September 1 – June 30), according to the specific school attended by each participant. The completeness of daily exposure data for each aggregation period was assessed, and in cases where completeness was below 50%, the aggregated exposure was set to missing in subsequent analyses to minimize exposure misclassification. The temporal adjustment method assumes stability in the relative spatial patterns of pollution over time, an assumption supported by the ESCAPE methodology and the high year-to-year correlations observed at the selected monitoring stations [16].

### Assessment of neurodevelopment outcome

At 4 years of age, children underwent individualized cognitive assessments conducted by trained psychologists. Children IQ and cognitive abilities were assessed by the Spanish version of the Wechsler Preschool and Primary Scale of Intelligence—fourth edition (WPPSI-IV) [22]. The WPPSI-IV is summarized into primary indexes, secondary indexes, and a full-scale IQ (FSIQ) score. For primary indexes, we obtained the Verbal Comprehension Index (VCI—reflects language development, vocabulary level, and verbal reasoning), the Fluid Reasoning Index (FRI—assess the child’s ability to identify patterns and relationships and think logically), the Working Memory Index (WMI—measures the child’s ability to hold, manipulate, and recall information over short periods to complete cognitive tasks), and the processing speed index (PSI—reflects the child’s ability to quickly and accurately scan, discriminate, and sequence simple visual information). Secondary indexes were also calculated: the vocabulary acquisition index (VAI—measures verbal learning and language development), the nonverbal index (NVI—includes child’s ability to solve problems, reason, and understand concepts using visual and hands-on tasks, without relying on language skills), and general ability index (GAI—measures cognitive ability emphasizing verbal and nonverbal reasoning while minimizing the influence of working memory and processing speed). All index scores are standardized to a mean of 100 and a standard deviation of 15.

### Assessment of covariates

Analyses were adjusted for maternal and child covariates selected based on previous studies demonstrating their potential influence on child neurodevelopment [14, 23, 24]. Maternal characteristics were collected through structured questionnaires administered during in-person interviews at enrollment and included maternal age, body mass index (BMI, kg/m^2^), smoking status (never smoker, ex-smoker, or current smoker), and socioeconomic status. Family social class was derived by combining parental occupational status, coded according to the Catalan Classification of Occupations (CCO-2011) [25], and parental educational level, and was categorized as low, medium, or high. Parental intelligence was assessed using the Matrix Reasoning subtest of the Wechsler Adult Intelligence Scale–III (WAIS-III) [26]. Both mothers and fathers completed the assessment, and the mean of the two scaled scores was used to approximate overall parental cognitive background. Child characteristics included gestational age at birth, obtained from the babies’ health cards, sex, and type of feeding as reported by the mother. In addition, residential greenness was assessed using the normalized difference vegetation index (NDVI) within a 500 m buffer around the school.

### Statistical analysis

#### Participants and characteristics

Participant characteristics were described using counts and percentages for categorical variables and mean ± standard deviation (SD) for continuous variables.

### Exposures

Air pollution exposures were standardized (z-scores). Regression coefficients represent the association per 1-SD increase in each pollutant, allowing direct comparability across pollutants. Spearman correlation was applied to examine pairwise relationships among air pollution exposures [27].

### Outcomes

Neurodevelopmental outcomes were assessed using the eight WPPSI cognitive indices (VCI, FRI, WMI, PSI, FSIQ, VAI, NVI, GAI). All indices were analyzed simultaneously to comprehensively evaluate cognitive development.

### Covariates

Covariates were selected based on prior literature and clinical relevance [28–31]. Specifically, maternal age, BMI, smoking status, family social class, and residential greenness (NDVI) were adjusted as potential confounders. Furthermore, parental IQ, child gestational age, and type of feeding were included as precision variables to reduce residual variance and improve the precision of the effect estimates for air pollution. Child sex was excluded from sex-stratified analyses.

### Imputation and statistical models

Missing data were addressed using multiple imputation by chained equations (MICE) under the missing at random (MAR) assumption. All exposures, covariates, and outcomes were included as predictors in the imputation models to improve imputation accuracy and ensure model congeniality. Outcome values themselves were not imputed, and analyses were performed only among participants with observed outcomes. Five imputed datasets were generated. Regression models were fitted separately in each imputed dataset, and the resulting estimates and standard errors were pooled using Rubin’s rules to obtain final effect estimates and 95% confidence intervals [32]. All analyses were conducted in the multiply imputed dataset (*n* = 286). Sample size did not vary across outcomes. Sex-stratified models included 140 females and 146 males.

Generalized additive models (GAMs) were employed to explore potential non-linear associations between air pollution exposure and neurodevelopmental outcomes. Smoothing parameters were automatically selected via Restricted Maximum Likelihood (REML) within *mgcv* package, ensuring an appropriate balance between model complexity and data fit [33]. Multivariable linear regression models were subsequently conducted to estimate the linear effects of individual exposures and sex interactions. Weighted quantile sum (WQS) used the *gwqs* package with repeated holdout bootstrap, which incorporates a training/testing split in each bootstrap iteration (validation = 0.6) to estimate mixture weights while minimizing overfitting. We performed 1000 bootstrap iterations (*b* = 1000) and allowed for both positive and negative associations [34]. Additionally, two-index WQS approach was applied to separately evaluate the positive and negative mixture effects on the outcome. Both linear regression and WQS analyses were further stratified by child sex to investigate potential sex-specific associations.

The statistical significance level was set at α = 0.05 and all statistical tests were two-sided.

## Results

### Characteristics of the study population

A comparison of characteristics between included and excluded participants was shown in Supplementary Table 1. Table 1 presents the included child characteristics together with cognitive outcomes and environmental exposures, as well as maternal and family characteristics (*n* = 286). Cognitive performance assessed using the WPPSI revealed mean scores ranging from 95.7 to 104.9 on the FSIQ and primary index scales (VCI, FRI, WMI, PSI), and from 97.5 to 105.9 on the Secondary Index Scales (VAI, NVI, GAI). Regarding air pollution, the mean concentrations were 27.1 ± 9.9 μg/m^3^ for NO_2_, 45.4 ± 17.7 μg/m^3^ for NOₓ, 11.0 ± 1.5 μg/m^3^ for PM_2.5_, 24.2 ± 3.3 μg/m^3^ for PM_10_, 14.0 ± 3.2 μg/m^3^ for PM_coarse_, 1.5 ± 0.4 × 10⁻^5^ m⁻^1^ for PM2.5_abs_, and 66.7 ± 3.7 μg/m^3^ for O_3_.

**Table 1:** Maternal and child characteristics, cognitive function scores, and environmental exposures (*n* = 286)

	Summary statistics	Missing *n* (%)
Children characteristics		
Gestational age (weeks), mean ± SD	39.8 ± 1.4	2 (0.7%)
Sex, *n* (%)		0 (0%)
Male	146 (51.0)	
Female	140 (49.0)	
Type of feeding, *n* (%)		0 (0.0%)
Breastfeeding	222 (77.6)	
Mixed feeding/infant formula	64 (22.4)	
NDVI within 500 m of school	0.3 (0.1)	35 (12.2%)
Maternal and family characteristics		
Age (years), mean ± SD	31.6 ± 4.7	0 (0%)
BMI, mean ± SD	24.7 ± 4.3	0 (0%)
Social class, *n* (%)		0 (0%)
Low	29 (10.1)	
Medium	189 (66.1)	
High	68 (23.8)	
Smoking status, *n* (%)		0 (0%)
Never smoker	198 (69.2)	
Smoker or ex-smoker	88 (30.8)	
Parent IQ (score), mean ± SD	9.1 ± 3.6	8 (2.8%)
Cognitive development (WPPSI score) 0 (0%)		
Primary index scales		
VCI	104.9 ± 13.4	
FRI	102.7 ± 13.1	
WMI	98.0 ± 12.1	
PSI	95.7 ± 12.7	
FSIQ	102.3 ± 11.7	
Secondary index scales		
VAI	97.5 ± 14.0	
NVI	101.2 ± 12.2	
GAI	105.9 ± 12.1	
Air pollution 35 (12.2%)		
NO_2_, μg/m^3^	27.1 ± 9.9	
NO_x_, μg/m^3^	45.4 ± 17.7	
PM_2.5_, μg/m^3^	11.0 ± 1.5	
PM_2.5abs,_ 10^−5^ m^−1^	1.5 ± 0.4	
PM_10_, μg/m^3^	24.2 ± 3.3	
PM_coarse_, μg/m^3^	14.0 ± 3.2	
O_3_, μg/m^3^	66.7 ± 3.7	

Abbreviations: *BMI* body mass index, *NDVI* normalized difference vegetation index, *WPPSI* Wechsler Preschool and Primary Scale of Intelligence, *VCI* verbal comprehension index, *FRI* fluid reasoning index, *WMI* working memory index, *PSI* processing speed index, *FSIQ* full scale IQ, *VAI* vocabulary acquisition index, *NVI* nonverbal index, *GAI* general ability index, *NO*_*2*_ nitrogen dioxide, *NO*_*x*_ nitrogen oxides, *PM*_2.5_ particulate matter ≤ 2.5 μm in diameter, *PM*_*2.5*_*abs* absorbance of particulate matter ≤ 2.5 μm, *PM*_*10*_ particulate matter ≤ 10 μm in diameter, *PM*_*coarse*_ coarse particulate matter (PM_10_ − PM_2.5_), *O*_3_ ozone

Figure S3 shows the Spearman correlations among air pollutants. Strong positive correlations were observed between NO_2_ and PM_2.5abs_ (*ρ* = 0.98), NO_x_ and NO_2_ (*ρ* = 0.97), and NO_x_ and PM_2.5abs_ (*ρ* = 0.97). PM₁₀ was moderately correlated with PM_coarse_ (*ρ* = 0.85), NO_x_ (*ρ* = 0.79), NO_2_ (*ρ* = 0.81), and PM_2.5abs_ (*ρ* = 0.79), while correlations among other pollutants were generally low.

### The association between air pollution and cognitive function scores

GAMs were first applied to assess potential non-linear associations between air pollutants and cognitive function scores. All smooth terms were linear (effective degrees of freedom = 1), supporting the use of linear regression models. Formal interaction tests including exposure × sex terms did not indicate statistically significant effect modification by sex (p range from 0.074 to 0.975) (Supplementary Table 2); therefore, sex-stratified results were presented for descriptive purposes only.

**Fig. 1:**
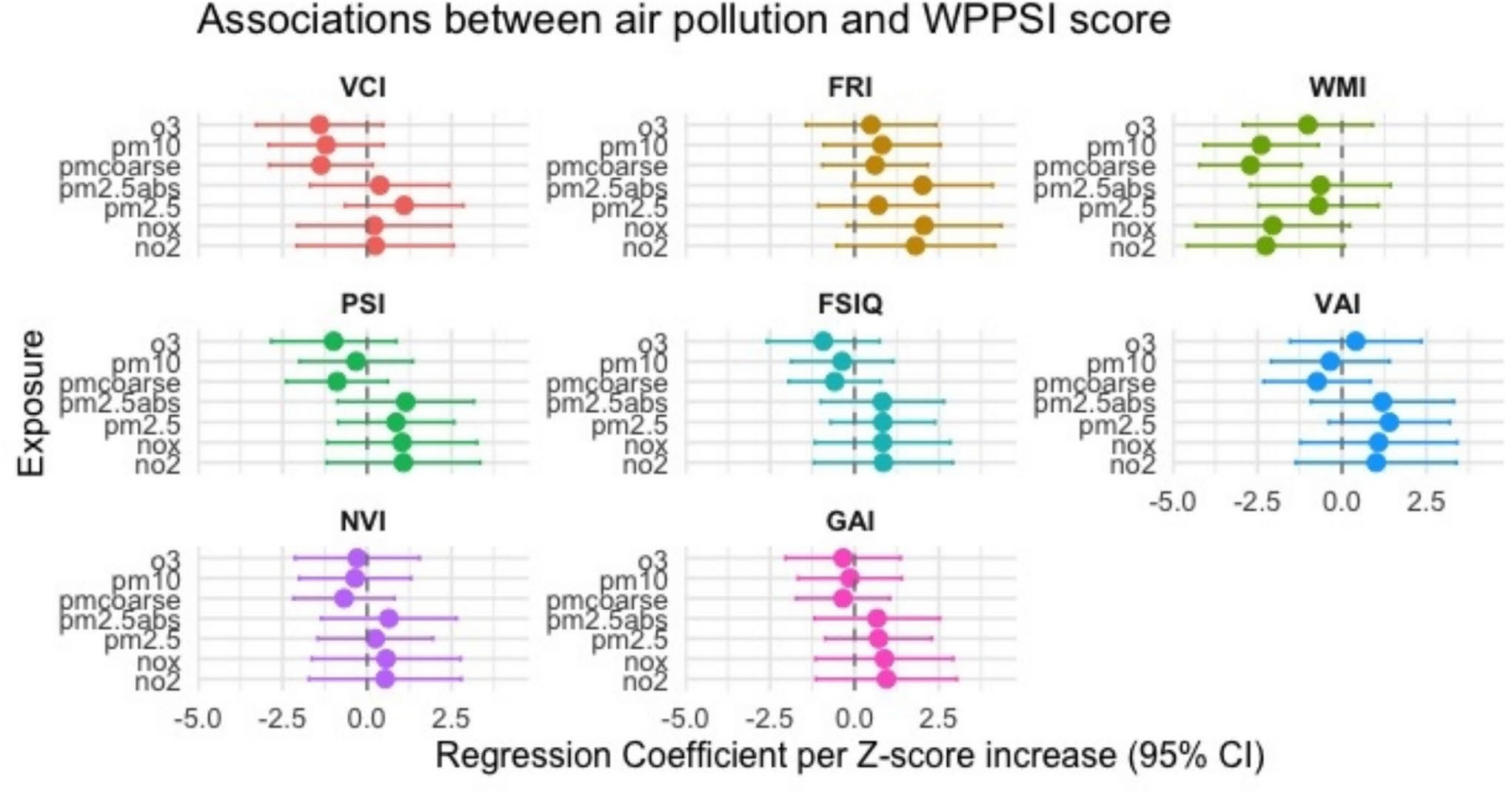
Multivariable linear regression of individual air pollutants with cognitive function scores. VCI, verbal comprehension index; FRI, fluid reasoning index; WMI, working memory index; PSI, processing speed index; FSIQ, full scale IQ; VAI, vocabulary acquisition index; NVI, nonverbal index; GAI, general ability index; NO_2_, nitrogen dioxide; NO_x_, nitrogen oxides; PM_2.5_, particulate matter ≤ 2.5 μm in diameter; PM_2.5_abs, absorbance of particulate matter ≤ 2.5 μm; PM_10_, particulate matter ≤ 10 μm in diameter; PM_coarse_, coarse particulate matter (PM_10_ − PM_2.5_); O_3_, ozone

In multivariable linear regression analyses, higher exposure to PM_coarse_ (*β* = −2.71, 95% confidence interval (CI): −4.23, −1.20) and PM_10_ (*β* = −2.39, 95% CI −4.09, −0.70) were associated with lower WMI in overall population (Fig. 1). After false discovery rate (FDR) adjustment for multiple testing, the association for PMcoarse remained statistically significant (*p* = 0.030), while the association for PM10 did not (*p* = 0.170). Full regression results including raw *p*-values and FDR-adjusted *p*-values are provided in Supplementary Table 3.

**Fig. 2:**
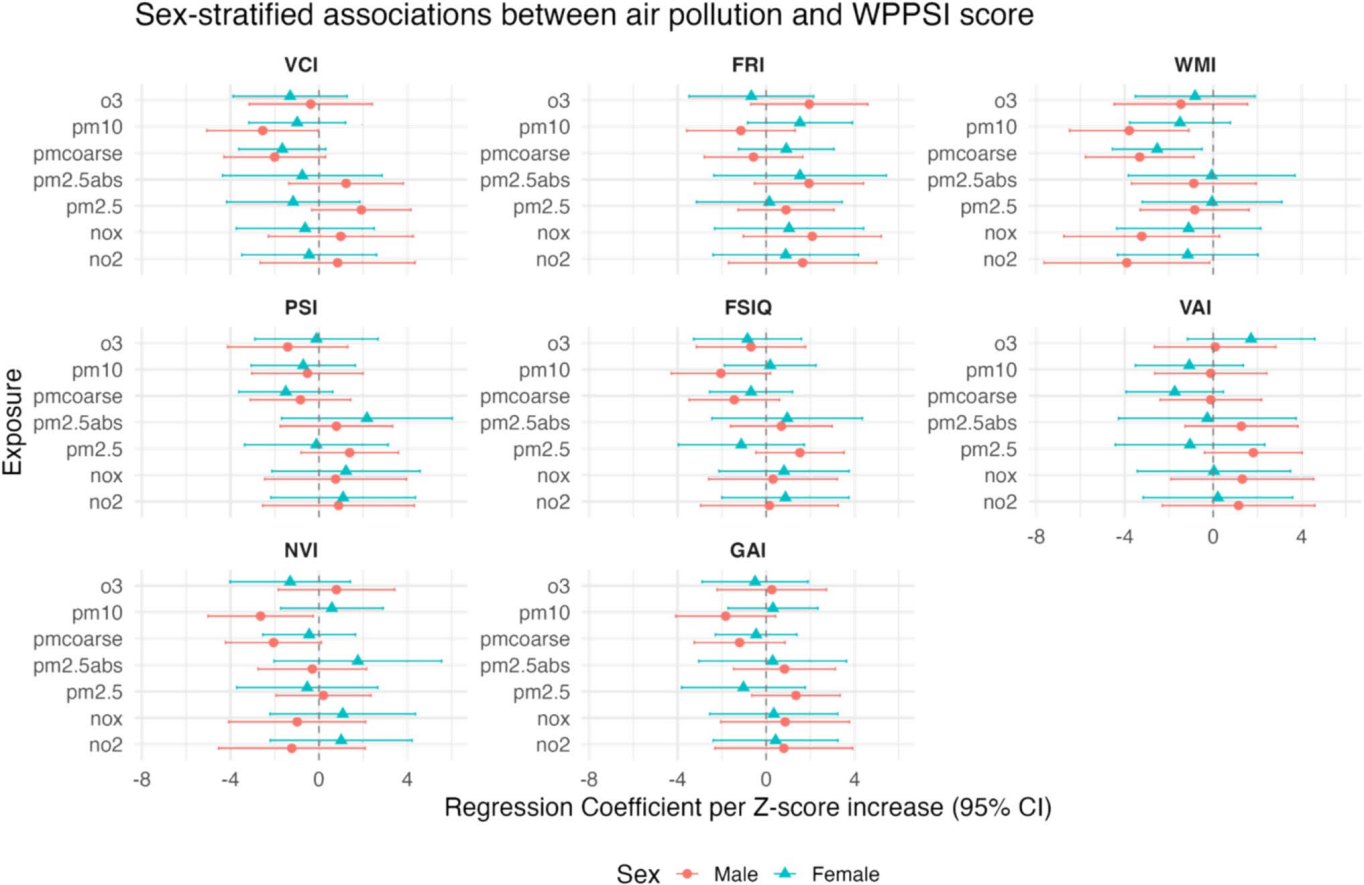
Multivariable linear regression of individual air pollutants with cognitive function scores stratified by sex. VCI, verbal comprehension index; FRI, fluid reasoning index; WMI, working memory index; PSI, processing speed index; FSIQ, full scale IQ; VAI, vocabulary acquisition index; NVI, nonverbal index; GAI, general ability index; NO_2_, nitrogen dioxide; NO_x_, nitrogen oxides; PM_2.5_, particulate matter ≤ 2.5 μm in diameter; PM_2.5_abs, absorbance of particulate matter ≤ 2.5 μm; PM_10_, particulate matter ≤ 10 μm in diameter; PM_coarse_, coarse particulate matter (PM_10_ − PM_2.5_); O_3_, ozone

Sex-stratified analyses revealed inverse associations predominantly in boys. Specifically, higher exposure to PM_10_ was associated with lower VCI scores (*β* = − 2.54, 95% CI − 5.07, − 0.02). In addition, negative associations with WMI were observed for NO₂ (*β* = − 3.91, 95% CI − 7.65, − 0.16), PM_10_ (*β* = − 3.79, 95% CI − 6.50, − 1.09), and PM_coarse_ (*β* = − 3.32, 95% CI − 5.77, − 0.88). PM_10_ exposure was also inversely associated with NVI in boys (*β* = − 2.64, 95% CI − 5.02, − 0.26). Among girls, an inverse association was observed between PM_coarse_ exposure and WMI (*β* = − 2.53, 95% CI − 4.56, − 0.50) (Fig. 2). Detailed sex-stratified regression results are shown in Supplementary Table 4. After FDR adjustment for multiple testing, none of the sex-stratified associations remained statistically significant (data not shown).

### The association between pollution mixture and cognitive function scores

**Fig. 3:**
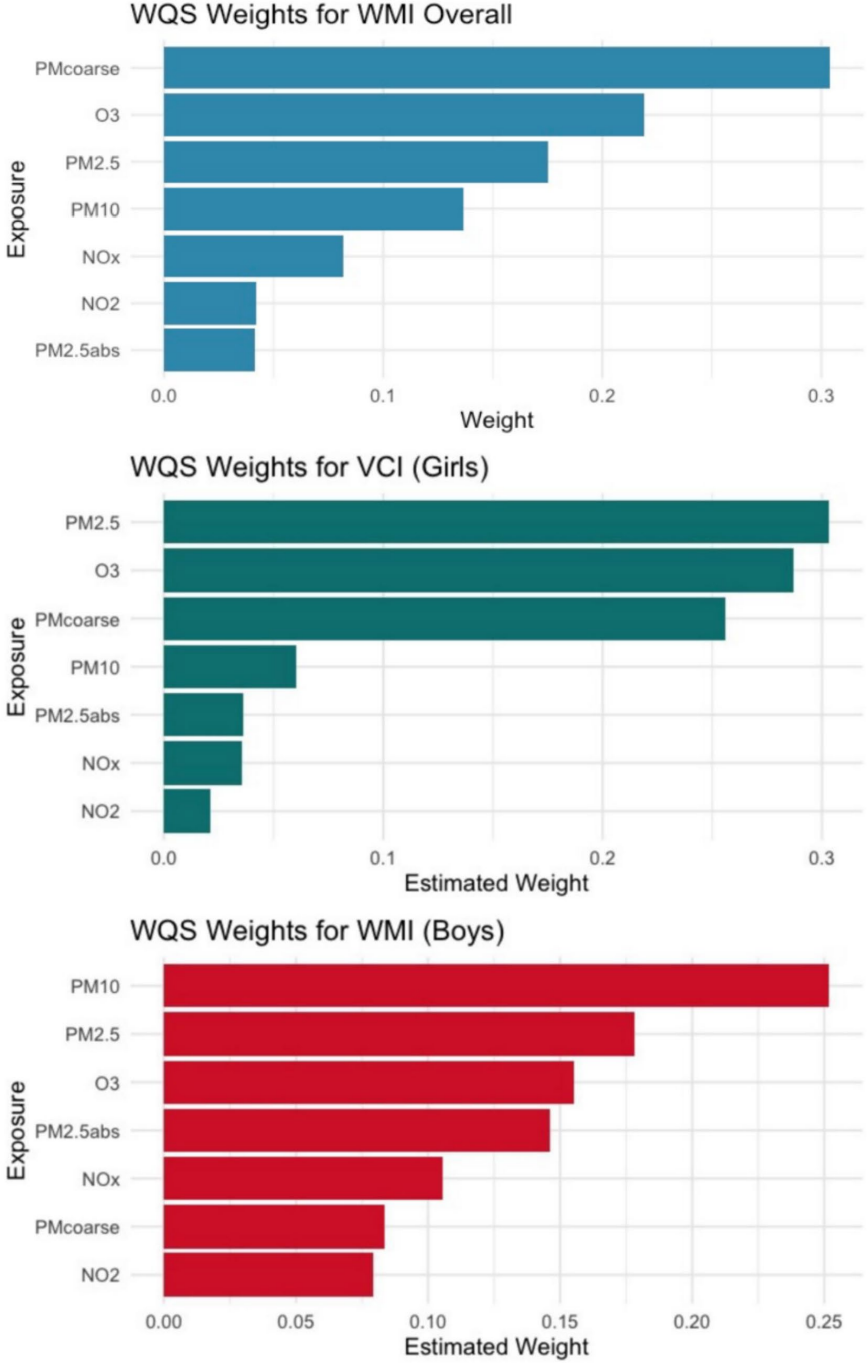
Contributions of individual air pollutants to the overall association with working memory index in all population, verbal comprehension index in girls and working memory index in boys by weighted quantile sum. WMI, working memory index; NO_2_, nitrogen dioxide; NO_x_, nitrogen oxides; PM_2.5_, particulate matter ≤ 2.5 μm in diameter; PM_2.5_abs, absorbance of particulate matter ≤ 2.5 μm; PM_10_, particulate matter ≤ 10 μm in diameter; PM_coarse_, coarse particulate matter (PM_10_ − PM_2.5_); O_3_, ozone

The overall association between air pollution mixture and WPPSI scores was examined. Results showed that the mixture had a negative association with WMI (estimate: −3.60, 95% CI −5.92, −1.28) (Table 2). When stratified by sex, air pollution negatively associated with VCI among girls (estimate: −2.90, 95% CI −4.69, −1.10) and WMI among boys (estimate: −5.39, 95% CI −9.30, −1.47) (Table 3). In all population, PM_coarse_ was the largest contributor. In girls, PM_2.5_, O_3_, and PM_coarse_ were the main contributors, whereas in boys, PM_10_ was the primary contributors (Fig. 3). WQS regression was additionally performed in the positive direction, and the previously observed associations in the negative direction were not observed (Supplementary Tables 5 & 6). Additionally, two-indices WQS model was applied to account for potential bidirectional effects of the mixture. In this model, neither the positive nor negative index reached statistical significance (Supplementary Table 7).

**Table 2:** The overall effect of air pollution mixture on WPPSI scores by WQS

	Estimate	95%CI
VCI	−1.02	−3.57, 1.52
FRI	0.38	−3.13, 3.89
WMI	−3.60*	−**5.92, **−1.28*
PSI	−2.03	−3.94, −0.12
FSIQ	0.43	−1.91, 2.79
VAI	1.11	−1.35, 3.59
NVI	−0.65	−4.02, 2.72
GAI	0.02	−2.86, 2.91

Abbreviations: *WPPSI* Wechsler Preschool and Primary Scale of Intelligence, *VCI* verbal comprehension index, *FRI* fluid reasoning index, *WMI* working memory index, *PSI* processing speed index, *FSIQ* full scale IQ, *VAI* vocabulary acquisition index, *NVI* nonverbal index, *GAI* general ability index

The cognitive proficiency index (CPI) is not shown separately as it is derived from WMI and PSI. The index represents the combined effect of multiple pollutants, with each pollutant’s concentration quartile weighted by its estimated contribution. Coefficients are interpreted as the effect per unit increase in the WQS index. *, statistical significant

**Table 3:** Overall effect of air pollution mixture on WPPSI scores: sex-stratified WQS analysis

		Estimate	95%CI
VCI	Boys	1.30	−2.62, 5.23
	Girls	−2.90*	−**4.69, **−1.10*
FRI	Boys	2.28	−1.98, 6.54
	Girls	0.39	−2.96, 3.76
WMI	Boys	−5.39*	−**9.30, **−1.47*
	Girls	−1.93	−5.61, 1.75
PSI	Boys	−0.72	−3.78, 2.33
	Girls	0.64	−3.88, 5.16
FSIQ	Boys	3.98	0.81, 7.15
	Girls	−0.73	−4.79, 3.33
VAI	Boys	4.22	−0.15, 8.60
	Girls	−0.24	−3.91, 3.42
NVI	Boys	2.85	−1.64, 7.34
	Girls	0.88	−3.22, 4.99
GAI	Boys	0.73	−2.88, 4.35
	Girls	−0.69	−4.15, 2.76

Abbreviations: *WPPSI* Wechsler Preschool and Primary Scale of Intelligence, *VCI* verbal comprehension index, *FRI* fluid reasoning index, *WMI* working memory index, *PSI* processing speed index, *FSIQ* full scale IQ, *VAI* vocabulary acquisition index, *NVI* nonverbal index, *GAI* general ability index

The index represents the combined effect of multiple pollutants, with each pollutant’s concentration quartile weighted by its estimated contribution. Coefficients are interpreted as the effect per unit increase in the WQS index. *, statistical significant

## Discussion

In this study, higher exposure to ambient air pollution was generally associated with lower cognitive function scores in preschool children. In single-pollutant analyses, PM_coarse_ and PM_10_ were negatively associated with working memory, with descriptive trends suggesting stronger associations in boys. In analyses of the pollution mixture, overall exposure was also negatively associated with working memory, with PM_coarse_ contributing most prominently. Sex-stratified trends suggested boys tended to show lower working memory, whereas girls tended to show lower verbal comprehension. Overall, these findings indicate that both individual pollutants and combined exposure are associated with lower cognitive function, with working memory appearing particularly sensitive.

Our study adds to the growing corpus of international evidence indicating a negative association between particulate matter exposure and cognitive development in children, such as the consistent associations reported for particulate matter with working memory and other executive functions [7, 14, 24]. Echoing findings from cohorts like the BREATHE project [8], we found that exposure to PM_coarse_ and PM_10_ was negatively associated with working memory scores. This is a crucial extension of the existing literature, which has predominantly focused on fine particles [35, 36], as it suggests that larger particulates from sources like road dust and construction may also penetrate indoor environments and threaten the developing brain [37]. Notably, strong correlations were observed among several traffic-related pollutants, reflecting their shared combustion sources and complicating the identification of independent effects [38]. To address this and to account for the reality that humans are exposed to pollutant mixtures rather than single agents, we applied WQS regression to assess joint association [34]. WQS generates a single mixture index that stabilizes effect estimates under conditions of high collinearity; however, the weights assigned to individual components should be interpreted as indicators of their relative contribution within the mixture rather than as independent or causal effects. Within this framework, the WQS analysis confirmed an overall negative association between the pollutant mixture and working memory, with PM_coarse_ emerging as the predominant contributor. Among executive functions, working memory is particularly crucial in children, as it enables them to temporarily hold and manipulate information, thereby supporting learning, problem-solving, and behavior regulation [6]. Working memory is also the executive function most strongly related to academic performance, especially in reading and mathematics [5, 39]. Therefore, the observed negative association of PM_coarse_ with working memory underscores not only a neurodevelopmental concern but also potential downstream consequences on children’s academic outcomes and psychosocial functioning. Unlike PM_2.5_, which can penetrate deep into the alveoli and enter the systemic circulation [40], coarse particles might be primarily deposited in the upper airways [41]. This could trigger a cascade involving the release of pro-inflammatory cytokines (e.g., IL-1β, TNF-α) into circulation [42], potentially compromising the blood–brain barrier, activating microglia, and causing neuroinflammation that disrupts key processes like hippocampal synaptogenesis and myelination which can associate with the working memory [2]. An alternative or complementary route could be direct translocation via the olfactory nerve [43]. Thus, our research underscores PM_coarse_ as a potent and potentially under-appreciated component of the neurotoxic air pollution mixture, urging a shift in the public health conversation beyond PM_2.5_ and toward more complex exposure scenarios.

Contrary to the pattern observed in the overall cohort, stratification by sex uncovered a more complex picture. In girls, the single-pollutant models showed an inverse association was observed between PM_coarse_ exposure and WMI. However, the WQS mixture analysis revealed a novel association: exposure to the pollutant mixture was linked to lower scores in the verbal comprehension score. Furthermore, the primary drivers of this association were PM_2.5_, O_3_, and PM_coarse_. These sex-specific patterns may reflect underlying developmental trajectories: girls tend to show more advanced language and verbal skills in early childhood, which might render verbal comprehension more sensitive to environmental insults such as air pollution [44]. This striking result suggests that girls may be susceptible to a different pollutant profile, including particulate and gaseous pollutants impacting distinct neural pathways, potentially related to language and verbal reasoning networks. Mechanisms could involve endocrine disruption or differential inflammatory responses to smaller particles and gaseous pollutants, highlighting a previously overlooked environmental risk factor for neurodevelopment in girls [45, 46]. In boys, PM_10_ was negatively associated with multiple cognitive domains, including verbal and non-verbal abilities as well as working memory, suggesting a broad impact on cognitive function. WQS mixture analysis confirmed these findings, showing that PM_10_ was the primary contributor to the association with working memory. Notably, boys may show greater susceptibility to environmental insults from pregnancy, potentially due to sex-specific neurodevelopmental processes and hormonal influences that persist beyond early neurodevelopment [47–50]. This increased susceptibility could explain why the mixture association with working memory was more pronounced in boys [8]. However, given the correlated nature of the exposure mixture and the interpretational constraints inherent to WQS derived weights, these sex-specific patterns should be viewed as suggestive rather than definitive evidence of causal effects. Although our stratified analyses indicated potential variations in cognitive responses between males and females, these findings remain strictly descriptive and hypothesis-generating because formal interaction tests did not reach statistical significance. Consequently, these observations require further validation in larger cohorts powered to detect true effect modification. Additionally, two-indices WQS analysis did not yield statistically significant results for either index, which suggested that partitioning the mixture may have reduced statistical power or reflected a degree of directional sensitivity within this sample. Nevertheless, since the initial unidirectional results exhibited the most coherent association, they remain the primary focus of our discussion, with the two-indices model serving as a conservative diagnostic of weight stability.

Our study possesses several notable strengths. First, the application of both single-pollutant models and multi-pollutant mixture approaches (WQS regression) provides a more realistic and comprehensive assessment of real-world exposure association. While the former helps identify individual signals, the latter effectively captures the combined neurotoxicity of the pollutant mixture and identifies the key contributors within it, thereby overcoming some limitations of traditional regression models in handling high collinearity [34]. Second, our sex-stratified analysis moves beyond simply reporting differential effects to hypothesizing distinct pathogenic pathways, which offered a nuanced perspective for future mechanistic research. Third, our findings highlight the importance of considering both coarse and fine particulate matter in assessing neurodevelopmental risks. Finally, the use of robust outcome measurements, with cognitive development assessed using the WPPSI, a validated instrument widely used in early childhood research, strengthens the reliability of our findings.

Several limitations should be considered. The cross-sectional design precludes causal inference. Air pollution exposure was assessed at the school level using ambient estimates, without personal monitoring, indoor air quality data, or information on exposures at home or other locations, which may have resulted in exposure misclassification. While exposure was assessed only at school age and relied on temporal adjustment assumptions that may not fully capture spatial–temporal variability, this school-level assignment did not account for variations in school attendance patterns (e.g., school moves or late entry), which may have introduced selection bias. Additionally, these estimates for pollutants without pollutant-specific temporal data are best characterized as semi-quantitative rankings, reflecting relative exposure contrasts between participants rather than precise absolute values. Although we adjusted for major child- and maternal-level covariates, unmeasured school-specific characteristics and other potential covariates identified in prior studies could not be accounted for, and residual confounding remains possible. We evaluated multiple correlated cognitive outcomes derived from the WPPSI test battery, which increases the possibility of chance findings due to multiple testing; therefore, the results should be interpreted with caution. Given the exploratory nature of our study, we applied FDR; the key association between PM_coarse_ and working memory remained robust in the overall population, while sex-stratified associations were not significant, highlighting the need for cautious interpretation. In addition, although sex–exposure interactions were formally tested and none reached statistical significance, sex-stratified analyses were conducted for descriptive purposes only. Analyses were restricted to children with complete cognitive outcome data, which reduced the sample size and may have introduced selection bias. Finally, exposure assignment at the school level did not account for within-school clustering, which may have affected the precision of the estimates.

In summary, our study provides evidence that real-world exposure to air pollution mixtures at school is associated with sex-specific deficits in cognitive development in young children. The key finding is a divergence in vulnerability: boys’ working memory is more associated, while girls’ verbal comprehension is more associated. This underscores the necessity of examining pollutant mixtures and conducting sex-stratified analyses to fully understand the neurodevelopmental risks posed by air pollution. Protecting children’s cognitive health requires public health strategies that address these complex exposure profiles and differential vulnerabilities.

## Supplementary Information

Below is the link to the electronic supplementary material.

ESM 1 (DOCX 410 KB)

## Data Availability

The data, variable definitions, and R scripts used in this study are available on reasonable request from the corresponding author.

## Abbreviations

BMI: Body mass index
CI: Confidence interval
FDR: False discovery rate
FISQ: Full-scale intelligence quotient
FRI: Fluid reasoning index
GAM: Generalized additive model
GAI: General ability index
IQ: Intelligence quotient
LUR: Land use regression
NDVI: Normalized difference vegetation index
NO_2_: Nitrogen dioxide
NO_x_: Nitrogen oxides
NVI: Nonverbal index
O_3_: Ozone
PM: Particulate matter
PM_2.5_: Particulate matter ≤ 2.5 μm in diameter
PM_2.5_abs: Absorbance of particulate matter ≤ 2.5 μm
PM_10_: Particulate matter ≤ 10 μm in diameter
PM_coarse_: Coarse particulate matter (PM_10_− PM_2.5_)
PSI: Processing speed index
SD: Standard deviation
VAI: Vocabulary acquisition index
VCI: Verbal comprehension index
WMI: Working memory index
WPPSI-IV: Wechsler Preschool and Primary Scale of Intelligence–Fourth Edition
WQS: Weighted quantile sum

## References

[1] Rentschler J, Leonova N (2023) Global air pollution exposure and poverty. Nat Commun 14:4432. 10.1038/s41467-023-39797-4 37481598 10.1038/s41467-023-39797-4 PMC10363163

[2] Ehsanifar M, Yavari Z, Rafati M (2022) Exposure to urban air pollution particulate matter: neurobehavioral alteration and hippocampal inflammation. Environ Sci Pollut Res 29:50856–50866. 10.1007/s11356-022-19367-9 10.1007/s11356-022-19367-9 35237914

[3] Jayaraj RL, Rodriguez EA, Wang Y, Block ML (2017) Outdoor ambient air pollution and neurodegenerative diseases: the neuroinflammation hypothesis. Curr Environ Health Rep 4:166–179. 10.1007/s40572-017-0142-3 28444645 10.1007/s40572-017-0142-3

[4] Guxens M, Lubczyńska MJ, Muetzel RL et al (2018) Air pollution exposure during fetal life, brain morphology, and cognitive function in school-age children. Biol Psychiatry 84:295–303. 10.1016/j.biopsych.2018.01.016 29530279 10.1016/j.biopsych.2018.01.016

[5] Carpentier P, Morneau-Vaillancourt G, Aubé S et al (2022) A sequential model of the contribution of preschool fluid and crystallized cognitive abilities to later school achievement. PLoS One 17:e0276532. 10.1371/journal.pone.0276532 36399469 10.1371/journal.pone.0276532 PMC9674147

[6] Ruffini C, Berni M, Pierucci G, Pecini C (2024) Executive functions as predictors of learning prerequisites in preschool: a longitudinal study. Trends Neurosci Educ 36:100239. 10.1016/j.tine.2024.100239 39266119 10.1016/j.tine.2024.100239

[7] Clifford A, Lang L, Chen R et al (2016) Exposure to air pollution and cognitive functioning across the life course – a systematic literature review. Environ Res 147:383–398. 10.1016/j.envres.2016.01.018 26945620 10.1016/j.envres.2016.01.018

[8] Sunyer J, Esnaola M, Alvarez-Pedrerol M et al (2015) Association between traffic-related air pollution in schools and cognitive development in primary school children: a prospective cohort study. PLoS Med 12:e1001792. 10.1371/journal.pmed.1001792 25734425 10.1371/journal.pmed.1001792 PMC4348510

[9] Castagna A, Mascheroni E, Fustinoni S, Montirosso R (2022) Air pollution and neurodevelopmental skills in preschool- and school-aged children: a systematic review. Neurosci Biobehav Rev 136:104623. 10.1016/j.neubiorev.2022.104623 35331818 10.1016/j.neubiorev.2022.104623

[10] Black MM, Walker SP, Fernald LCH et al (2017) Early childhood development coming of age: science through the life course. Lancet 389:77–90. 10.1016/S0140-6736(16)31389-7 27717614 10.1016/S0140-6736(16)31389-7 PMC5884058

[11] Lin C-C, Yang S-K, Lin K-C et al (2014) Multilevel analysis of air pollution and early childhood neurobehavioral development. Int J Environ Res Public Health 11:6827–6841. 10.3390/ijerph110706827 24992486 10.3390/ijerph110706827 PMC4113847

[12] Ha S, Yeung E, Bell E et al (2019) Prenatal and early life exposures to ambient air pollution and development. Environ Res 174:170–175. 10.1016/j.envres.2019.03.064 30979514 10.1016/j.envres.2019.03.064 PMC6541527

[13] Guilbert A, Bernard JY, Peyre H et al (2023) Prenatal and childhood exposure to ambient air pollution and cognitive function in school-age children: examining sensitive windows and sex-specific associations. Environ Res 235:116557. 10.1016/j.envres.2023.116557 37423370 10.1016/j.envres.2023.116557

[14] Gui Z, Cai L, Zhang J et al (2020) Exposure to ambient air pollution and executive function among Chinese primary schoolchildren. Int J Hyg Environ Health 229:113583. 10.1016/j.ijheh.2020.113583 32917369 10.1016/j.ijheh.2020.113583

[15] Arija V, Fargas F, March G et al (2014) Adapting iron dose supplementation in pregnancy for greater effectiveness on mother and child health: protocol of the ECLIPSES randomized clinical trial. BMC Pregnancy Childbirth 14:33. 10.1186/1471-2393-14-33 24438754 10.1186/1471-2393-14-33 PMC3898489

[16] Cyrys J, Eeftens M, Heinrich J et al (2012) Variation of NO2 and NOx concentrations between and within 36 European study areas: results from the ESCAPE study. Atmos Environ 62:374–390. 10.1016/j.atmosenv.2012.07.080

[17] Eeftens M, Tsai M-Y, Ampe C et al (2012) Spatial variation of PM2.5, PM10, PM2.5 absorbance and PMcoarse concentrations between and within 20 European study areas and the relationship with NO2 – results of the ESCAPE project. Atmos Environ 62:303–317. 10.1016/j.atmosenv.2012.08.038

[18] Beelen R, Hoek G, Vienneau D et al (2013) Development of NO2 and NOx land use regression models for estimating air pollution exposure in 36 study areas in Europe – the ESCAPE project. Atmos Environ 72:10–23. 10.1016/j.atmosenv.2013.02.037

[19] de Hoogh K, Chen J, Gulliver J et al (2018) Spatial PM2.5, NO2, O3 and BC models for Western Europe – evaluation of spatiotemporal stability. Environ Int 120:81–92. 10.1016/j.envint.2018.07.036 30075373 10.1016/j.envint.2018.07.036

[20] de Hoogh K, Wang M, Adam M et al (2013) Development of land use regression models for particle composition in twenty study areas in Europe. Environ Sci Technol 47:5778–5786. 10.1021/es400156t 23651082 10.1021/es400156t

[21] Wang M, Beelen R, Basagana X et al (2013) Evaluation of land use regression models for NO _2_ and particulate matter in 20 European study areas: the ESCAPE project. Environ Sci Technol 47:4357–4364. 10.1021/es305129t 23534892 10.1021/es305129t

[22] Watkins MW, Beaujean AA (2014) Bifactor structure of the Wechsler Preschool and Primary Scale of Intelligence--Fourth Edition. Sch Psychol Q 29(1):52–63. 10.1037/spq0000038 10.1037/spq0000038 24188289

[23] Meador KJ, Baker GA, Browning N et al (2011) Relationship of child IQ to parental IQ and education in children with fetal antiepileptic drug exposure. Epilepsy Behav 21:147–152. 10.1016/j.yebeh.2011.03.020 21546316 10.1016/j.yebeh.2011.03.020 PMC3114203

[24] Forns J, Dadvand P, Esnaola M et al (2017) Longitudinal association between air pollution exposure at school and cognitive development in school children over a period of 3.5 years. Environ Res 159:416–421. 10.1016/j.envres.2017.08.031 28858754 10.1016/j.envres.2017.08.031

[25] De Catalunya G (2011) Classificació catalana d’ocupacions (CCO-2011). Adaptació de la CNO-2011. https://www.idescat.cat/metodes/classificacions/cco-2011-ca?lang=es

[26] Ryan JJ, Lopez SJ (2001) Wechsler Adult Intelligence Scale-III. Understanding psychological assessment. Springer, US, Boston, MA, pp 19–42

[27] Akoglu H (2018) User’s guide to correlation coefficients. Turk J Emerg Med 18:91–93. 10.1016/j.tjem.2018.08.001 30191186 10.1016/j.tjem.2018.08.001 PMC6107969

[28] Goulding N, Northstone K, Taylor CM et al (2025) Differences in neurocognitive development between children who had had no breast milk and those who had had breast milk for at least 6 months. Nutrients 17:2847. 10.3390/nu17172847 40944234 10.3390/nu17172847 PMC12430489

[29] Nivins S, Padilla N, Kvanta H, Ådén U (2025) Gestational age and cognitive development in childhood. JAMA Netw Open 8:e254580. 10.1001/jamanetworkopen.2025.4580 40227687 10.1001/jamanetworkopen.2025.4580 PMC11997729

[30] Pezzuti L, Farese M, Dawe J, Lauriola M (2025) The role of parental education, intelligence, and personality on the cognitive abilities of gifted children. J Intell 13:12. 10.3390/jintelligence13020012 39997163 10.3390/jintelligence13020012 PMC11856753

[31] Opbroek J, Pereira Barboza E, Nieuwenhuijsen M et al (2024) Urban green spaces and behavioral and cognitive development in children: a health impact assessment of the Barcelona “Eixos Verds” Plan (Green Axis Plan). Environ Res 244:117909. 10.1016/j.envres.2023.117909 38103780 10.1016/j.envres.2023.117909

[32] Tang F, Ishwaran H (2017) Random forest missing data algorithms. Statistical Analysis and Data Mining: The ASA Data Science Journal 10:363–377. 10.1002/sam.11348 10.1002/sam.11348 PMC5796790 29403567

[33] Wood SN (2011) Fast stable restricted maximum likelihood and marginal likelihood estimation of semiparametric generalized linear models. J R Stat Soc Ser B Stat Methodol 73:3–36. 10.1111/j.1467-9868.2010.00749.x

[34] Renzetti S, Gennings C, Calza S (2023) A weighted quantile sum regression with penalized weights and two indices. Front Public Health. 10.3389/fpubh.2023.1151821 37533534 10.3389/fpubh.2023.1151821 PMC10392701

[35] Lertxundi A, Andiarena A, Martínez MD et al (2019) Prenatal exposure to PM2.5 and NO2 and sex-dependent infant cognitive and motor development. Environ Res 174:114–121. 10.1016/j.envres.2019.04.001 31055169 10.1016/j.envres.2019.04.001

[36] Chang Y-C, Chen W-T, Su S-H et al (2022) PM2.5 exposure and incident attention-deficit/hyperactivity disorder during the prenatal and postnatal periods: a birth cohort study. Environ Res 214:113769. 10.1016/j.envres.2022.113769 35777438 10.1016/j.envres.2022.113769

[37] Zhang Y, Jia Z, Rajendran RS et al (2021) Exposure of particulate matter (PM10) induces neurodevelopmental toxicity in zebrafish embryos. Neurotoxicology 87:208–218. 10.1016/j.neuro.2021.10.004 34678400 10.1016/j.neuro.2021.10.004

[38] Vienneau D, Stafoggia M, Rodopoulou S et al (2023) Association between exposure to multiple air pollutants, transportation noise and cause-specific mortality in adults in Switzerland. Environ Health 22:29. 10.1186/s12940-023-00983-y 36967400 10.1186/s12940-023-00983-y PMC10041702

[39] Sankalaite S, Huizinga M, Warreyn P et al (2023) The association between working memory, teacher-student relationship, and academic performance in primary school children. Front Psychol. 10.3389/fpsyg.2023.1240741 37809289 10.3389/fpsyg.2023.1240741 PMC10556679

[40] Thangavel P, Park D, Lee Y-C (2022) Recent insights into particulate matter (PM2.5)-mediated toxicity in humans: an overview. Int J Environ Res Public Health 19:7511. 10.3390/ijerph19127511 35742761 10.3390/ijerph19127511 PMC9223652

[41] Brunekreef B, Forsberg B (2005) Epidemiological evidence of effects of coarse airborne particles on health. Eur Respir J 26:309–318. 10.1183/09031936.05.00001805 16055881 10.1183/09031936.05.00001805

[42] Brook RD, Rajagopalan S, Pope CA et al (2010) Particulate matter air pollution and cardiovascular disease. Circulation 121:2331–2378. 10.1161/CIR.0b013e3181dbece1 20458016 10.1161/CIR.0b013e3181dbece1

[43] Amato-Lourenço LF, Dantas KC, Júnior GR et al (2024) Microplastics in the olfactory bulb of the human brain. JAMA Netw Open 7:e2440018. 10.1001/jamanetworkopen.2024.40018 39283733 10.1001/jamanetworkopen.2024.40018 PMC11406405

[44] Rinaldi P, Pasqualetti P, Volterra V, Caselli MC (2023) Gender differences in early stages of language development. Some evidence and possible explanations. J Neurosci Res 101:643–653. 10.1002/jnr.24914 34240751 10.1002/jnr.24914

[45] Seli DA, Taylor HS (2023) The impact of air pollution and endocrine disruptors on reproduction and assisted reproduction. Curr Opin Obstet Gynecol 35:210–215. 10.1097/GCO.0000000000000868 36924404 10.1097/GCO.0000000000000868

[46] Parenteau AM, Hang S, Swartz JR et al (2024) Clearing the air: a systematic review of studies on air pollution and childhood brain outcomes to mobilize policy change. Dev Cogn Neurosci 69:101436. 10.1016/j.dcn.2024.101436 39244820 10.1016/j.dcn.2024.101436 PMC11407021

[47] Liao Y-C, Xu Y-J, Chen J-K et al (2023) Sex differences in children’s cognitive functions and phthalates exposure: a meta-analysis. Pediatr Res 94:1609–1618. 10.1038/s41390-023-02672-5 37264138 10.1038/s41390-023-02672-5 PMC10624603

[48] Moore SE (2024) Sex differences in growth and neurocognitive development in infancy and early childhood. Proc Nutr Soc 83:221–228. 10.1017/S0029665124000144 38326969 10.1017/S0029665124000144

[49] Goodman CV, Green R, DaCosta A et al (2023) Sex difference of pre- and post-natal exposure to six developmental neurotoxicants on intellectual abilities: a systematic review and meta-analysis of human studies. Environ Health 22:80. 10.1186/s12940-023-01029-z 37978510 10.1186/s12940-023-01029-z PMC10655280

[50] Chen B, Huang S, He J et al (2020) Sex-specific influence of prenatal air pollutant exposure on neonatal neurobehavioral development and the sensitive window. Chemosphere 254:126824. 10.1016/j.chemosphere.2020.126824 32335443 10.1016/j.chemosphere.2020.126824

