# Supplementary material for: Air pollution mixtures and cognitive outcomes in children: associations with school-age exposure and sex differences
Source: Eur J Pediatr. 2026 Mar 19;185(4):192. doi: 10.1007/s00431-026-06841-6 (PMC13002747; doi:10.1007/s00431-026-06841-6)
Supplement: Supplementary file 1 — (DOCX 410 KB) [file 431_2026_6841_MOESM1_ESM.docx]

| 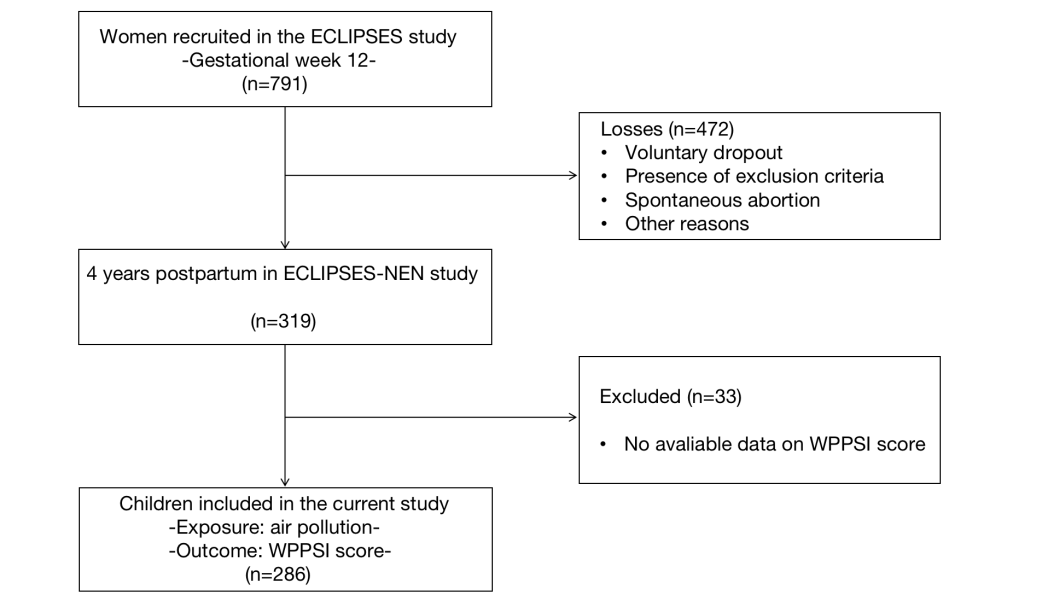  **Fig. S1** Study flow diagram.  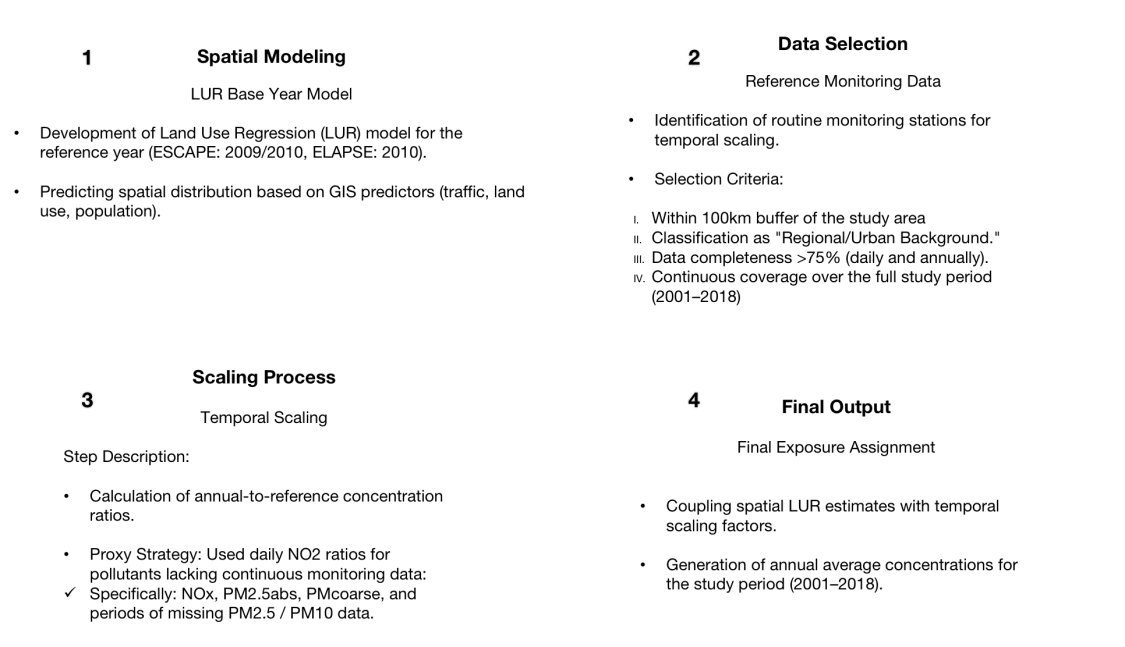  **Fig. S2** Schematic of the LUR Temporal Adjustment and Exposure Assignment Process.  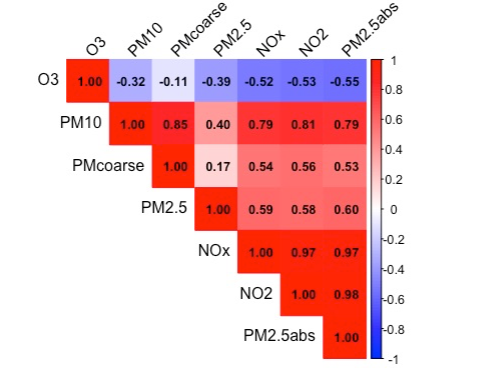  **Fig. S3** Spearman correlations among air pollutants  Supplementary Table 1. Comparison of characteristics between included and excluded participants | | | |
| --- | --- | --- | --- |
| Children characteristics | Excluded | Included | P |
| Gestational age (weeks), mean ± SD | 39.7 ± 1.4 | 39.8 ± 1.4 | 0.453 |
| Sex, n (%) |  |  | 0.472 |
| Male | 244 (48.3) | 146 (51.0) |  |
| Female | 261 (51.7) | 140 (49.0) |  |
| Type of feeding, n (%) |  |  | 0.940 |
| Breastfeeding | 390 (77.2) | 222 (77.6) |  |
| Mixed feeding/infant formula | 115 (22.8) | 64 (22.4) |  |
| NDVI within 500 m of school | 0.3 ± 0.1 | 0.3 ± 0.1 | 0.681 |
| Maternal and family characteristics | | |  |
| Age (years), mean ± SD | 31.0 ± 5.2 | 31.6 ± 4.7 | 0.090 |
| BMI, mean ± SD | 24.7 ± 4.3 | 24.7 ± 4.3 | 0.870 |
| Social class, n (%) |  |  | 0.260 |
| Low | 66 (13.1%) | 29 (10.1) |  |
| Medium | 339 (67.1%) | 189 (66.1) |  |
| High | 100 (19.8%) | 68 (23.8) |  |
| Smoking status, n (%) |  |  | 0.450 |
| Never smoker | 335 (66.3) | 198 (69.2) |  |
| Smoker or ex-smoker | 170 (33.7) | 88 (30.8) |  |
| Parent IQ (score), mean ± SD | 8.9 ± 2.7 | 9.1 ± 3.6 | 0.350 |
| Abbreviations: BMI, Body Mass Index; MedDiet, Mediterranean diet; NDVI, Normalized Difference Vegetation Index. | | | |
|  | | | |

| Supplementary table 2. The interaction between sex and air pollution | | | | | | | | |
| --- | --- | --- | --- | --- | --- | --- | --- | --- |
|  | VCI | FRI | WMI | PSI | FSIQ | VAI | NVI | GAI |
| Air pollution | b (95%CI) | b (95%CI) | b (95%CI) | b (95%CI) | b (95%CI) | b (95%CI) | b (95%CI) | b (95%CI) |
| NO2 | 1.02 (-1.81, 3.85) | 0.62 (-2.35, 3.59) | 1.77 (-1.04, 4.59) | 0.29 (-2.49, 3.06) | 1.43 (-1.14, 4.00) | 0.21 (-2.67, 3.10) | 1.07 (-1.72, 3.85) | 0.56 (-2.03, 3.14) |
| *p* | 0.480 | 0.681 | 0.217 | 0.839 | 0.273 | 0.884 | 0.453 | 0.672 |
| NO_x_ | 0.29 (-2.55, 3.13) | 0.93 (-2.05, 3.91) | 1.35 (-1.52, 4.22) | 0.64 (-2.14, 3.43) | 1.37 (-1.22, 3.97) | 0.11 (-2.79, 3.01) | 0.93 (-1.90, 3.76) | 0.82 (-1.79, 3.43) |
| *p* | 0.843 | 0.538 | 0.356 | 0.649 | 0.298 | 0.940 | 0.516 | 0.538 |
| O_3_ | -0.74 (-3.58, 2.10) | -2.10 (-5.12, 0.92) | -0.43 (-3.30, 2.43) | 0.20 (-2.60, 3.00) | -0.63 (-3.23, 1.97) | 0.64 (-2.29, 3.58) | -1.01 (-3.88, 1.86) | -1.34 (-3.99, 1.31) |
| *p* | 0.608 | 0.173 | 0.767 | 0.889 | 0.636 | 0.666 | 0.488 | 0.321 |
| PM_10_ | 1.64 (-1.17, 4.46) | 2.63 (-0.33, 5.60) | 2.17 (-0.61, 4.95) | -0.56 (-3.34, 2.22) | 1.47 (-1.10, 4.04) | -0.18 (-3.07, 2.71) | 2.53 (-0.25, 5.31) | 1.51 (-1.08, 4.09) |
| *p* | 0.252 | 0.082 | 0.126 | 0.694 | 0.262 | 0.903 | 0.074 | 0.253 |
| PM_25_ | -2.12 (-5.08, 0.84) | -0.05 (-3.26, 3.15) | 0.78 (-2.44, 4.00) | 0.06 (-2.95, 3.07) | -0.53 (-3.39, 2.33) | -0.53 (-3.68, 2.63) | -0.94 (-4.13, 2.26) | -1.24 (-4.11, 1.63) |
| *p* | 0.160 | 0.975 | 0.635 | 0.967 | 0.714 | 0.742 | 0.565 | 0.397 |
| PM_2.5abs_ | -1.27 (-4.19, 1.64) | 1.86 (-1.30, 5.03) | 1.17 (-2.17, 4.51) | 1.98 (-1.00, 4.95) | 1.37 (-1.41, 4.14) | 0.30 (-2.72, 3.33) | 1.08 (-2.09, 4.26) | 1.03 (-1.83, 3.88) |
| *p* | 0.390 | 0.247 | 0.492 | 0.192 | 0.333 | 0.844 | 0.503 | 0.479 |
| PM_coarse_ | 0.32 (-2.52, 3.17) | 1.42 (-1.58, 4.41) | 0.87 (-1.90, 3.63) | -1.69 (-4.47, 1.08) | 0.15 (-2.45, 2.74) | -1.15 (-4.04, 1.73) | 0.97 (-1.84, 3.78) | -0.22 (-2.81, 2.38) |
| *p* | 0.823 | 0.353 | 0.537 | 0.231 | 0.912 | 0.433 | 0.496 | 0.869 |
| Abbreviations: WPPSI, Wechsler Preschool and Primary Scale of Intelligence; VCI, Verbal Comprehension Index; FRI, Fluid Reasoning Index; WMI, Working Memory Index; PSI, Processing Speed Index; FSIQ, Full Scale IQ; VAI, Vocabulary Acquisition Index; NVI, Nonverbal Index; GAI, General Ability index; edf, effective degrees of freedom. NO_2_, Nitrogen Dioxide; NOₓ, Nitrogen Oxides; PM_2.5_, Particulate Matter ≤2.5 µm in diameter; PM_2.5abs_, Absorbance of Particulate Matter ≤2.5 µm; PM_10_, Particulate Matter ≤10 µm in diameter; PM_coarse_, Coarse Particulate Matter (PM_10_ − PM_2.5_); O_3_, Ozone. | | | | | | | | |

| Supplementary table 3. Associations between air pollutant exposures and cognitive function scores, including Raw and False Discovery Rate (FDR)-adjusted p-values. | | | | |
| --- | --- | --- | --- | --- |
| Outcome | Exposure | Beta_95CI | Raw_P | FDR_P |
| FRI | no2 | 1.81 (-0.54, 4.15) | 0.132 | 0.674 |
| FRI | nox | 2.06 (-0.23, 4.34) | 0.079 | 0.648 |
| FRI | pm25 | 0.70 (-1.08, 2.48) | 0.441 | 0.674 |
| FRI | pm25abs | 2.01 (-0.06, 4.09) | 0.059 | 0.648 |
| FRI | pmcoarse | 0.61 (-0.95, 2.17) | 0.445 | 0.674 |
| FRI | pm10 | 0.81 (-0.92, 2.54) | 0.358 | 0.674 |
| FRI | o3 | 0.49 (-1.44, 2.41) | 0.621 | 0.787 |
| FSIQ | no2 | 0.85 (-1.20, 2.91) | 0.416 | 0.674 |
| FSIQ | nox | 0.83 (-1.18, 2.84) | 0.419 | 0.674 |
| FSIQ | pm25 | 0.84 (-0.71, 2.39) | 0.292 | 0.674 |
| FSIQ | pm25abs | 0.82 (-1.00, 2.65) | 0.378 | 0.674 |
| FSIQ | pmcoarse | -0.59 (-1.95, 0.77) | 0.398 | 0.674 |
| FSIQ | pm10 | -0.37 (-1.88, 1.14) | 0.634 | 0.787 |
| FSIQ | o3 | -0.92 (-2.59, 0.74) | 0.279 | 0.674 |
| GAI | no2 | 0.95 (-1.14, 3.03) | 0.375 | 0.674 |
| GAI | nox | 0.88 (-1.16, 2.92) | 0.396 | 0.674 |
| GAI | pm25 | 0.71 (-0.87, 2.29) | 0.378 | 0.674 |
| GAI | pm25abs | 0.67 (-1.19, 2.52) | 0.482 | 0.710 |
| GAI | pmcoarse | -0.34 (-1.73, 1.04) | 0.628 | 0.787 |
| GAI | pm10 | -0.14 (-1.68, 1.40) | 0.859 | 0.859 |
| GAI | o3 | -0.34 (-2.04, 1.36) | 0.696 | 0.787 |
| NVI | no2 | 0.54 (-1.72, 2.79) | 0.641 | 0.787 |
| NVI | nox | 0.56 (-1.64, 2.77) | 0.616 | 0.787 |
| NVI | pm25 | 0.24 (-1.47, 1.95) | 0.781 | 0.825 |
| NVI | pm25abs | 0.64 (-1.36, 2.65) | 0.529 | 0.760 |
| NVI | pmcoarse | -0.68 (-2.18, 0.81) | 0.371 | 0.674 |
| NVI | pm10 | -0.36 (-2.01, 1.30) | 0.674 | 0.787 |
| NVI | o3 | -0.29 (-2.14, 1.56) | 0.759 | 0.817 |
| PSI | no2 | 1.07 (-1.20, 3.35) | 0.355 | 0.674 |
| PSI | nox | 1.03 (-1.19, 3.26) | 0.362 | 0.674 |
| PSI | pm25 | 0.86 (-0.86, 2.58) | 0.328 | 0.674 |
| PSI | pm25abs | 1.14 (-0.88, 3.16) | 0.268 | 0.674 |
| PSI | pmcoarse | -0.89 (-2.40, 0.61) | 0.246 | 0.674 |
| PSI | pm10 | -0.33 (-2.00, 1.35) | 0.703 | 0.787 |
| PSI | o3 | -0.99 (-2.84, 0.86) | 0.296 | 0.674 |
| VAI | no2 | 1.01 (-1.37, 3.39) | 0.405 | 0.674 |
| VAI | nox | 1.08 (-1.24, 3.41) | 0.361 | 0.674 |
| VAI | pm25 | 1.40 (-0.39, 3.20) | 0.127 | 0.674 |
| VAI | pm25abs | 1.20 (-0.92, 3.31) | 0.269 | 0.674 |
| VAI | pmcoarse | -0.74 (-2.32, 0.84) | 0.360 | 0.674 |
| VAI | pm10 | -0.35 (-2.10, 1.41) | 0.699 | 0.787 |
| VAI | o3 | 0.41 (-1.54, 2.35) | 0.683 | 0.787 |
| VCI | no2 | 0.24 (-2.08, 2.57) | 0.838 | 0.859 |
| VCI | nox | 0.21 (-2.07, 2.48) | 0.859 | 0.859 |
| VCI | pm25 | 1.10 (-0.65, 2.85) | 0.220 | 0.674 |
| VCI | pm25abs | 0.38 (-1.69, 2.44) | 0.722 | 0.793 |
| VCI | pmcoarse | -1.37 (-2.90, 0.16) | 0.081 | 0.648 |
| VCI | pm10 | -1.21 (-2.92, 0.49) | 0.164 | 0.674 |
| VCI | o3 | -1.41 (-3.28, 0.47) | 0.143 | 0.674 |
| WMI | no2 | -2.26 (-4.58, 0.07) | 0.059 | 0.648 |
| WMI | nox | -2.05 (-4.32, 0.23) | 0.079 | 0.648 |
| WMI | pm25 | -0.70 (-2.47, 1.08) | 0.442 | 0.674 |
| WMI | pm25abs | -0.64 (-2.72, 1.44) | 0.549 | 0.768 |
| WMI | pmcoarse | -2.71 (-4.23, -1.20) | 0.001* | 0.030* |
| WMI | pm10 | -2.39 (-4.09, -0.70) | 0.006* | 0.170 |
| WMI | o3 | -1.01 (-2.93, 0.90) | 0.302 | 0.674 |
| Abbreviations: FDR, False Discovery Rate; VCI, Verbal Comprehension Index; FRI, Fluid Reasoning Index; WMI, Working Memory Index; PSI, Processing Speed Index; FSIQ, Full Scale IQ; VAI, Vocabulary Acquisition Index; NVI, Nonverbal Index; GAI, General Ability index; edf, effective degrees of freedom. NO_2_, Nitrogen Dioxide; NOₓ, Nitrogen Oxides; PM_2.5_, Particulate Matter ≤2.5 µm in diameter; PM_2.5abs_, Absorbance of Particulate Matter ≤2.5 µm; PM_10_, Particulate Matter ≤10 µm in diameter; PM_coarse_, Coarse Particulate Matter (PM_10_ − PM_2.5_); O_3_, Ozone. Raw P represents the unadjusted p-value from the regression models. FDR P represents the p-value adjusted for multiple comparisons using the Benjamini-Hochberg procedure. *, statistical significant | | | | |

| Supplementary table 4. Sex-stratified Association Between Air Exposure on WPPSI score | | | | | | | | | | | | | | | | |
| --- | --- | --- | --- | --- | --- | --- | --- | --- | --- | --- | --- | --- | --- | --- | --- | --- |
|  | VCI |  | FRI |  | WMI |  | PSI |  | FSIQ |  | VAI |  | NVI |  | GAI |  |
| Air pollution | b | 95%CI | b | 95%CI | b | 95%CI | b | 95%CI | b | 95%CI | b | 95%CI | b | 95%CI | b | 95%CI |
| **NO_2_** |  |  |  |  |  |  |  |  |  |  |  |  |  |  |  |  |
| boys | 0.84 (-2.66, 4.34) | | 1.65 (-1.69, 5.00) | | -3.91 (-7.65, -0.16)* | | 0.89 (-2.54, 4.31) | | 0.15 (-2.96, 3.25) | | 1.15 (-2.30, 4.59) | | -1.23 (-4.54, 2.08) | | 0.80 (-2.31, 3.91) | |
| girls | -0.45 (-3.49, 2.60) | | 0.89 (-2.40, 4.18) | | -1.15 (-4.33, 2.02) | | 1.09 (-2.18, 4.36) | | 0.88 (-1.99, 3.75) | | 0.21 (-3.17, 3.59) | | 1.01 (-2.20, 4.21) | | 0.43 (-2.39, 3.25) | |
| **NO_x_** |  |  |  |  |  |  |  |  |  |  |  |  |  |  |  |  |
| boys | 0.98 (-2.29, 4.26) | | 2.09 (-1.03, 5.21) | | -3.23 (-6.75, 0.29) | | 0.75 (-2.46, 3.96) | | 0.31 (-2.60, 3.22) | | 1.31 (-1.91, 4.54) | | -0.99 (-4.08, 2.11) | | 0.86 (-2.05, 3.77) | |
| girls | -0.63 (-3.74, 2.49) | | 1.04 (-2.33, 4.41) | | -1.11 (-4.36, 2.15) | | 1.22 (-2.13, 4.57) | | 0.81 (-2.13, 3.75) | | 0.03 (-3.43, 3.50) | | 1.08 (-2.21, 4.36) | | 0.35 (-2.54, 3.24) | |
| **O_3_** |  |  |  |  |  |  |  |  |  |  |  |  |  |  |  |  |
| boys | -0.37 (-3.16, 2.41) | | 1.95 (-0.69, 4.59) | | -1.46 (-4.48, 1.56) | | -1.41 (-4.13, 1.30) | | -0.68 (-3.15, 1.78) | | 0.09 (-2.66, 2.83) | | 0.79 (-1.84, 3.42) | | 0.26 (-2.21, 2.73) | |
| girls | -1.30 (-3.88, 1.27) | | -0.67 (-3.49, 2.15 | | -0.82 (-3.52, 1.88) | | -0.11 (-2.90, 2.67) | | -0.84 (-3.28, 1.60) | | 1.71 (-1.17, 4.59) | | -1.30 (-4.02, 1.42 | | -0.50 (-2.89, 1.90) | |
| **PM_10_** |  |  |  |  |  |  |  |  |  |  |  |  |  |  |  |  |
| boys | -2.54 (-5.07, -0.02)* | | -1.14 (-3.59, 1.31) | | -3.79 (-6.50, -1.09)* | | -0.52 (-3.03, 1.99) | | -2.04 (-4.29, 0.20) | | -0.11 (-2.64, 2.42) | | -2.64 (-5.02, -0.26)* | | -1.83 (-4.08, 0.43) | |
| girls | -0.98 (-3.17, 1.20) | | 1.53 (-0.84, 3.90) | | -1.50 (-3.78, 0.78) | | -0.71 (-3.07, 1.65) | | 0.19 (-1.88, 2.26) | | -1.08 (-3.52, 1.36) | | 0.58 (-1.73, 2.89) | | 0.31 (-1.72, 2.34) | |
| **PM_25_** |  |  |  |  |  |  |  |  |  |  |  |  |  |  |  |  |
| boys | 1.92 (-0.32, 4.15) | | 0.90 (-1.27, 3.07) | | -0.84 (-3.30, 1.63) | | 1.39 (-0.81, 3.60) | | 1.53 (-0.46, 3.52) | | 1.81 (-0.40, 4.02) | | 0.20 (-1.95, 2.35) | | 1.35 (-0.65, 3.35) | |
| girls | -1.16 (-4.18, 1.85) | | 0.15 (-3.15, 3.44) | | -0.05 (-3.21, 3.11) | | -0.12 (-3.37, 3.13) | | -1.12 (-3.97, 1.72) | | -1.05 (-4.43, 2.33) | | -0.53 (-3.72, 2.66) | | -1.02 (-3.82, 1.77) | |
| **PM_2.5abs_** |  |  |  |  |  |  |  |  |  |  |  |  |  |  |  |  |
| boys | 1.22 (-1.36, 3.81) | | 1.94 (-0.52, 4.40) | | -0.88 (-3.70, 1.94) | | 0.79 (-1.75, 3.33) | | 0.69 (-1.60, 2.99) | | 1.27 (-1.28, 3.82) | | -0.30 (-2.75, 2.16) | | 0.83 (-1.47, 3.13) | |
| girls | -0.75 (-4.36, 2.85) | | 1.54 (-2.36, 5.44) | | -0.07 (-3.84, 3.70) | | 2.17 (-1.69, 6.03) | | 0.96 (-2.45, 4.36) | | -0.27 (-4.28, 3.75) | | 1.76 (-2.03, 5.55) | | 0.30 (-3.05, 3.64) | |
| **PM_coarse_** |  |  |  |  |  |  |  |  |  |  |  |  |  |  |  |  |
| boys | -2.00 (-4.29, 0.29) | | -0.57 (-2.79, 1.66) | | -3.32 (-5.77, -0.88)* | | -0.84 (-3.11, 1.43) | | -1.44 (-3.48, 0.60) | | -0.11 (-2.40, 2.18) | | -2.05 (-4.22, 0.11) | | -1.20 (-3.25, 0.85) | |
| girls | -1.66 (-3.62, 0.31) | | 0.91 (-1.24, 3.07) | | -2.53 (-4.56, -0.50)* | | -1.50 (-3.62, 0.62) | | -0.68 (-2.55, 1.19) | | -1.74 (-3.94, 0.46) | | -0.44 (-2.54, 1.66) | | -0.45 (-2.29, 1.39) | |
| Abbreviations: WPPSI, Wechsler Preschool and Primary Scale of Intelligence; VCI, Verbal Comprehension Index; FRI, Fluid Reasoning Index; WMI, Working Memory Index; PSI, Processing Speed Index; FSIQ, Full Scale IQ; VAI, Vocabulary Acquisition Index; NVI, Nonverbal Index; GAI, General Ability index; edf, effective degrees of freedom. NO_2_, Nitrogen Dioxide; NOₓ, Nitrogen Oxides; PM_2.5_, Particulate Matter ≤2.5 µm in diameter; PM_2.5abs_, Absorbance of Particulate Matter ≤2.5 µm; PM_10_, Particulate Matter ≤10 µm in diameter; PM_coarse_, Coarse Particulate Matter (PM_10_ − PM_2.5_); O_3_, Ozone. *, statistical significant | | | | | | | | | | | | | | | | |

| Supplementary table 5. The overall effect of air pollution mixture on WPPSI scores by WQS in positive direction | | |
| --- | --- | --- |
|  | estimate | 95%CI |
| VCI | -1.69 | -4.21, 0.82 |
| FRI | 1.40 | -1.88, 4.70 |
| WMI | 1.86 | -4.11, 0.38 |
| PSI | 1.98 | -1.57, 5.55 |
| FSIQ | 0.59 | -1.79, 2.98 |
| VAI | 2.41 | 0.01, 4.81* |
| NVI | 0.16 | -2.35, 2.68 |
| GAI | -0.40 | -2.59, 1.77 |
| Abbreviations: WPPSI, Wechsler Preschool and Primary Scale of Intelligence; VCI, Verbal Comprehension Index; FRI, Fluid Reasoning Index; WMI, Working Memory Index; PSI, Processing Speed Index; FSIQ, Full Scale IQ; VAI, Vocabulary Acquisition Index; NVI, Nonverbal Index; GAI, General Ability index. The Cognitive Proficiency Index (CPI) is not shown separately as it is derived from WMI and PSI. *, statistical significant | | |

| Supplementary table 6. Overall effect of air pollution mixture on WPPSI scores: sex-stratified WQS analysis in opposite direction | | | |
| --- | --- | --- | --- |
|  |  | estimate | 95%CI |
| VCI | boys | 1.87 | -1.56, 5.30 |
|  | girls | -1.40 | -4.14, 1.33 |
| FRI | boys | 1.80 | -1.88, 5.50 |
|  | girls | 1.10 | -2.50, 4.70 |
| WMI | boys | -3.07 | -6.45, 0.29 |
|  | girls | -1.85 | -5.10, 1.40 |
| PSI | boys | 2.20 | -1.09, 5.50 |
|  | girls | 2.16 | -0.73, 5.07 |
| FSIQ | boys | -0.62 | -3.85, 2.60 |
|  | girls | -0.58 | -5.32, 4.14 |
| VAI | boys | 2.77 | -0.98, 6.52 |
|  | girls | 2.21 | -2.63, 7.07 |
| NVI | boys | -1.98 | -5.50, 1.52 |
|  | girls | 1.67 | -2.88, 6.23 |
| GAI | boys | 1.52 | -1.26, 5.90 |
|  | girls | -0.25 | -3.46, 2.95 |
| Abbreviations: WPPSI, Wechsler Preschool and Primary Scale of Intelligence; VCI, Verbal Comprehension Index; FRI, Fluid Reasoning Index; WMI, Working Memory Index; PSI, Processing Speed Index; FSIQ, Full Scale IQ; VAI, Vocabulary Acquisition Index; NVI, Nonverbal Index; GAI, General Ability index. | | | |

| Supplementary table 7. The overall effect of air pollution mixture on WPPSI scores by two-indices WQS | | |
| --- | --- | --- |
|  | estimate | 95%CI |
| VCI pwqs | 2.07 | -3.11, 7.26 |
| nwqs | -1.44 | -6.27, 3.37 |
| FRI pwqs | -0.01 | -3.64, 3.62 |
| nwqs | 0.91 | -3.08, 4.92 |
| WMI pwqs | 1.92 | -2.74, 6.59 |
| nwqs | -1.62 | -6.78, 3.52 |
| PSI pwqs | 4.15 | -0.60, 8.90 |
| nwqs | -4.18 | -9.31, 0.94 |
| FSIQ pwqs | 2.90 | -3.92, 9.73 |
| nwqs | -2.10 | -8.33, 4.11 |
| VAI pwqs | 0.95 | -3.54, 5.46 |
| nwqs | -0.67 | -5.25, 3.91 |
| NVI pwqs | 2.95 | 0.76, 5.14 |
| nwqs | -2.65 | -5.45, 0.12 |
| GAI pwqs | 2.20 | -3.46, 7.87 |
| nwqs | -1.46 | -6.41, 3.48 |
| Abbreviations: WPPSI, Wechsler Preschool and Primary Scale of Intelligence; VCI, Verbal Comprehension Index; FRI, Fluid Reasoning Index; WMI, Working Memory Index; PSI, Processing Speed Index; FSIQ, Full Scale IQ; VAI, Vocabulary Acquisition Index; NVI, Nonverbal Index; GAI, General Ability index. The Cognitive Proficiency Index (CPI) is not shown separately as it is derived from WMI and PSI. | | |
